# Patient Satisfaction with IBD Undergoing Colonoscopy: A Multicenter Cross-Sectional Study

**DOI:** 10.3390/jcm14082562

**Published:** 2025-04-08

**Authors:** Daniele Napolitano, Alessio Lo Cascio, Mattia Bozzetti, Arianna Povoli, Simonetta Grubissa, Luca Molino, Marco Marino, Debora Berretti, Pierluigi Puca, Diletta Immacolata Rita Lavigna, Fabio Grilli, Giulio Antonelli, Valentin Calvez, Amalia Di Petrillo, Sara Onali, Antonio Gasbarrini, Gionata Fiorino, Franco Scaldaferri

**Affiliations:** 1 CEMAD—Fondazione Policlinico Gemelli IRCCS, 00168 Rome, Italy; daniele.napolitano@policlinicogemelli.it (D.N.); dilettaimmacolatarita.lavigna@guest.policlinicogemelli.it (D.I.R.L.); fabio.grilli@policlinicogemelli.it (F.G.); valentino.calvez@gmail.com (V.C.); antonio.gasbarrini@unicatt.it (A.G.); franco.scaldaferri@policlinicogemelli.it (F.S.); 2 Department of Nursing Research and Management, La Maddalena Cancer Center, 90146 Palermo, Italy; locascio.alessio@lamaddalenanet.it; 3 Direction of Health Professions, ASST Cremona, 26100 Cremona, Italy; 4 Department of Gastroenterology, Azienda Sanitaria Universitaria Friuli Centrale, 33100 Udine, Italy; arianna.povoli@asufc.sanita.fvg.it (A.P.); simonetta.grubissa@asufc.sanita.fvg.it (S.G.); marco.marino@asufc.sanita.fvg.it (M.M.); debora.berretti@asufc.sanita.fvg.it (D.B.); 5 School of Medicine, Faculty of Medicine, Università Cattolica del Sacro Cuore, Largo Francesco Vito, 1, 00168 Rome, Italy; luca.molino01@icatt.it; 6 Gastroenterology Unit, Nuovo Regina Margherita Hospital, 00153 Rome, Italy; 7 Department of Medical Sciences and Public Health, University of Cagliari, 09142 Cagliari, Italy; amalia.dip@unica.it (A.D.P.); sara.onali@unica.it (S.O.); 8 Department of Medical and Surgical Sciences, Fondazione Policlinico Universitario A. Gemelli IRCCS, Università Cattolica del Sacro Cuore, 00168 Rome, Italy; 9 IBD Unit, Department of Gastroenterology and Digestive Endoscopy, San Camillo-Forlanini Hospital, 00152 Rome, Italy; gionataf@gmail.com

**Keywords:** inflammatory bowel diseases, patient satisfaction, quality of healthcare, endoscopy

## Abstract

**Background:** Colonoscopy is crucial for diagnosing and monitoring inflammatory bowel disease (IBD), assessing disease activity, and detecting dysplasia. However, patient adherence to surveillance remains suboptimal due to discomfort, anxiety, and concerns about bowel preparation. **Methods:** This multicenter cross-sectional study assessed patient satisfaction with colonoscopy in IBD patients across three Italian centers. Participants completed pre- and post-examination questionnaires, including the Endoscopy Customer Satisfaction Questionnaire (ECSQ) and Perceived Stress Scale (PSS-10). Clinical factors, bowel preparation methods, and healthcare provider expertise were analyzed. **Results:** Among 444 enrolled patients, overall satisfaction was high (98.8%) but varied across procedural phases. Higher satisfaction was predicted by expert endoscopists (β = 2.11, *p* = 0.012), disease remission (β = 1.70, *p* = 0.020), and frequent endoscopic procedures in the last 24 months (β = 0.46, *p* = 0.041). Conversely, severe disease activity (β = −3.87, *p* < 0.001) was associated with lower satisfaction. Deep sedation and high-volume bowel preparation negatively impacted satisfaction. **Conclusions:** Optimizing bowel preparation, enhancing healthcare provider expertise, and implementing stress-reducing strategies could improve patient adherence to surveillance guidelines in IBD care.

## 1. Introduction

Chronic colonoscopy plays a pivotal role in both the diagnosis and follow-up of Inflammatory Bowel Disease (IBD), enabling the early detection of dysplasia and colorectal cancer (CRC), as well as estimating disease activity and response to therapy. Despite its clinical importance, adherence to the recommended surveillance schedules remains suboptimal, often impeded by patient-related factors such as discomfort, anxiety, and negative perceptions of the procedure [1,2]. IBD’s incidence and prevalence are rapidly growing. The annual estimated number of new IBD cases in Italy is over 15,000 (about 55% being UC cases and 45% being CD); by 2030, the total number of IBD cases in Italy is expected to reach nearly 592,000. For this reason, optimizing resource management and improving the quality of diagnostics is of paramount importance for the correct handling of IBD patients and high-volume centers [3,4]. Previous studies have shown that patients frequently deviate from the recommended colonoscopy intervals due to perceived barriers, including discomfort, embarrassment, and anticipatory anxiety, as well as bowel preparation, which is often perceived as the most burdensome aspect of the procedure, albeit among the most important elements of a procedural quality. All these elements may be exacerbated in IBD patients, potentially undermining the effectiveness of IBD surveillance protocols [5,6].

The psychological impact of colonoscopy extends beyond immediate physical discomfort, encompassing the fear of the potential discovery of malignancies, procedural complications, and the overall invasiveness of the examination [7]. These fears are particularly pronounced in IBD patients who are already managing the complexities of a chronic disease [8,9,10,11].

Despite the recognized importance of patient satisfaction in IBD management [12], comprehensive data examining specific factors influencing satisfaction among IBD patients undergoing colonoscopy across multiple centers are limited. This gap in the literature hinders the development of tailored interventions that address the particular needs of distinct patient subgroups within the IBD population [13].

In this multicentric cross-sectional study, we aimed to comprehensively analyze patient satisfaction with colonoscopy among individuals with IBD.

This study aimed to comprehensively assess the level and determinants of patient satisfaction during colonoscopy among individuals with IBD in a multicentric setting.

## 2. Materials and Methods

### 2.1. Study Outcomes

The primary objective of this study was to assess the perceived satisfaction of IBD outpatients undergoing colonoscopy.

The secondary objectives are the following:

1. To examine how stress levels influence patient satisfaction, the expertise of endoscopists and nurses, disease activity, and various clinical factors (e.g., pathology type, surgical history, preparation methods, and sedation type).
2. To evaluate the determinants of patient satisfaction undergoing an endoscopic examination.

### 2.2. Design

This multicentric prospective cross-sectional study was conducted across multiple endoscopies in three endoscopy centers (Roma, Palermo, and Udine). Patients were recruited between December 2023 and September 2024 during their colonoscopies.

### 2.3. Participants

Consecutive IBD patients scheduled to undergo colonoscopy were enrolled in the study. After providing informed consent, they completed the research assessments described below. Participation was limited to the day of the examination, with no follow-up.

The inclusion criteria for patients were as follows: individuals aged 18 years and older who were seen at the outpatient Digestive Endoscopy services of the participating clinical centers for any indication for endoscopy related to IBD. Eligible patients included those with a confirmed diagnosis of IBD who could understand the Italian language, were not affected by debilitating mental illnesses, and were scheduled for a colonoscopy procedure.

Exclusion criteria included patients with documented colorectal cancer, pregnancy, significant respiratory impairment, and known psychiatric disorders that could interfere with providing informed consent or influence the perception of the procedure.

All participants provided written informed consent before data collection. All patients participated voluntarily and were informed about the study objectives, procedures, and their right to participate and withdraw from the study at any time. The participants provided informed consent and were given information about privacy protection. Furthermore, the participants’ data protection and anonymity were guaranteed by attributing a unique identification code.

### 2.4. Instruments

#### 2.4.1. Pre-Exam Patient Assessment

Administered via an oral interview by a trained endoscopy nurse upon patient arrival, specifically instructed on the standardized administration of the questionnaire and on patient communication. Demographic data, clinical history, and procedural details were collected through a structured 19-item pre-exam interview.

#### 2.4.2. Post-Exam Patient Assessment

This 5-item section, completed after the patient, recorded details on the type of anesthesia used (none, conscious, or deep sedation). It assessed the experience levels of both the nurse and the endoscopist (categorized as novice, competent, or expert according to established guidelines). Nurse competence was evaluated following the international guidelines; competence levels were categorized as novice (entry-level nurses with limited experience), competent (nurses with a solid background and practical experience), and expert (highly skilled nurses with advanced expertise and decision-making abilities in the field) [14,15,16]. Endoscopist expertise was categorized as “novice” for endoscopists in training, “competent” for those who have performed up to 500 completed procedures, and “expert” for those with over 500 completed procedures [17,18].

This assessment also recorded procedure completion, disease activity, and bowel cleanliness (via the Boston Bowel Preparation Scale) [19].

### 2.5. Endoscopy Customer Satisfaction Questionnaire (ECSQ)

Completed after the patient had fully regained consciousness (minimum five half-lives of anesthesia). The Endoscopy Customer Satisfaction Questionnaire (ECSQ) is a validated 18-item tool divided into four sections (Before, During, After, and Overall), and it uses a 5-point Likert scale for the first three sections and a dichotomous format for the Overall section [20]. The ECSQ showed excellent internal consistency (Cronbach’s α = 0.944) and was originally developed and validated in Italian endoscopy settings, as reported by Minciullo et al. [20].

Each section generates specific satisfaction scores as follows: Before the Examination (range 6–30), categorized as Low (6–14), Medium (15–22), or High (23–30); Examination (range 4–20), classified as Low (4–9), Medium (10–15), or High (16–20); and After the Examination (range 5–25), grouped as Low (5–12), Medium (13–19), or High (20–25). Total patient satisfaction was calculated by summing the scores from these three sections (range 15–75) and normalizing to a 0–100 scale. The total ECSQ score (range 15–75) was rescaled to a 0–100 scale using the following formula: (score − 15)/(75 − 15) × 100. Patients were also asked to respond to the following three dichotomous (yes/no) questions: “Were you overall satisfied with the treatment?”, “Did any issues occur on the day of the examination?”, and “Would you undergo another endoscopic procedure at the same facility?”. These questions aimed to assess overall satisfaction, the presence of any procedural concerns, and willingness to return for future examinations.

### 2.6. Perceived Stress Scale (PSS-10)

This tool, the Perceived Stress Scale (PSS-10) [21], measures perceived stress over the past month using a 5-point Likert scale, with four items reverse-scored. Total scores range from 0 to 40, classifying stress as low (0–13), moderate (14–26), or high (27–40). The PSS-10, validated in Italian, showed good reliability (Cronbach’s α ≈ 0.78–0.82) and was administered post-exam [22,23].

### 2.7. Disease Activity Assessment

For Ulcerative Colitis (UC), disease activity was evaluated using the partial Mayo score (clinical assessment) [24], inflammatory biomarkers (CRP ≤ 0.5 mg/dL and FCP ≤ 250 μg/g), and the Mayo endoscopic score. For Crohn’s Disease (CD), the assessment included the Harvey–Bradshaw Index, similar biomarker criteria, and the Simple Endoscopic Score for Crohn’s Disease (SES-CD) [25,26]. This integrated approach combined subjective symptoms with objective biomarkers and endoscopic findings.

### 2.8. Data Analysis

Statistical analyses were conducted on the dataset using descriptive statistical parameters. Initially, the normality of the distribution was verified through the Shapiro–Wilk and Kolmogorov–Smirnov tests, along with an assessment of kurtosis and skewness. Given the sensitivity of these tests to large sample sizes, visual inspections, such as Q-Q plots, were also conducted to provide additional context on distributional assumptions. Continuous variables were presented as the median and interquartile range (IQR), while categorical variables were expressed as frequencies and percentages. Correlations were examined using a bivariate Spearman correlation model. The Mann–Whitney U Test was applied for comparisons between two groups, while the Kruskal–Wallis ANOVA was used for analyses involving more than two groups. For multiple group comparisons, the ANOVA Dunn’s post hoc test was used to identify specific group differences. Bonferroni corrections were applied to adjust for multiple comparisons, reducing the risk of Type I errors and ensuring the robustness of the results. Chi-square (χ^2^) and Fisher’s exact test were used to examine associations between categorical variables. The Chi-square test was applied when sample sizes were sufficiently large, whereas Fisher’s exact test was used for smaller sample sizes or when the expected frequencies in any cell were below five, ensuring the robustness and accuracy of the association analyses.

To predict satisfaction, a robust regression model was implemented. The model was developed through a forward stepwise selection process, where predictors were added iteratively based on their statistical significance, ensuring the inclusion of variables that substantially enhanced the explained variance. The optimal model was determined using the following key evaluation metrics: the Akaike Information Criterion (AIC) to balance model fit and complexity, the Bayesian Information Criterion (BIC) to minimize overfitting, and computing Mc Fadden R2 to measure explanatory power while accounting for model size. This comprehensive approach ensured that the final model was both statistically robust and parsimonious.

### 2.9. Ethical Considerations

The study protocol was approved by the Lazio 2 Ethics Committee (pro. 258790/2023 del 30/11/2023).

All procedures performed in studies involving human participants complied with the ethical standards of the institutional and national research committee and with the 1964 Helsinki declaration and its later amendments or comparable ethical standards.

## 3. Results

**Figure 1:**
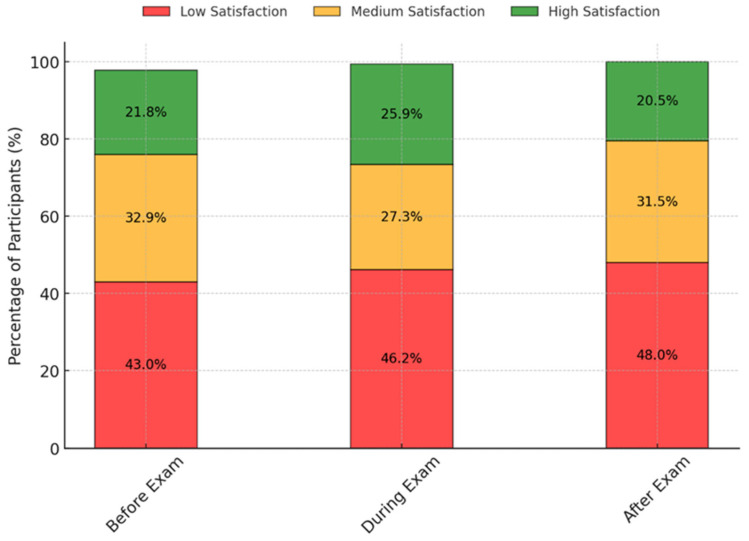
Percentage of participants who have reported ‘Low’, ‘Medium’, and ‘High’ satisfaction.

A total of 444 patients were enrolled in the study, with a balanced distribution between Crohn’s disease (56.5%) and ulcerative colitis (43.5%). The entire characteristics of the sample are reported in the Supplementary Materials and Table 1. As illustrated in Figure 1, satisfaction levels varied significantly across different phases of the examination process. Patient satisfaction with the endoscopic procedure was remarkably high. A total of 98.8% of patients reported being overall satisfied with their treatment experience. All patients reported that no issues occurred on the day of the examination and indicated that they would undergo another endoscopic procedure at the same facility in the future.

### 3.1. Relationship Between Patient Satisfaction and Stress Levels, Endoscopist and Nurse Expertise, Disease Activity, and Clinical Variables

A strong correlation emerged between satisfaction and stress levels. Higher satisfaction was consistently linked to lower perceived stress, as reflected in the significant negative correlations before (r = −0.18, *p* < 0.001) and during the procedure (r = −0.24, *p* < 0.001). Satisfaction levels before the exam were also positively correlated with those during (r = 0.79, *p* < 0.001) and after (r = 0.70, *p* < 0.001). These correlations suggest that lower perceived stress is consistently associated with higher satisfaction across the entire colonoscopy process. This underlines the potential value of implementing stress-reducing strategies to improve the patient experience and support adherence to surveillance protocols.

### 3.2. Relationship Between Patient Satisfaction and Healthcare Workers Competence

Examinations conducted by novice endoscopists resulted in lower satisfaction during the procedure (χ^2^(2) = 7.45, *p* = 0.024) compared to those performed by experienced or expert endoscopists. Although not statistically significant, a trend was observed in nurse experience

To investigate the influence of healthcare professionals’ expertise on patient satisfaction, a linear regression analysis was performed, considering the interaction between nurse and endoscopist competency levels. The model (R^2^ = 0.419) revealed that when both nurses and endoscopist were experts, patient satisfaction scores were significantly higher compared to other competency combinations (β = 5.69, *p* < 0.001). Similarly, the presence of an expert nurse, even when paired with a competent endoscopist, was associated with a positive but more modest increase in satisfaction (β = 3.43, *p* = 0.046). Conversely, examinations conducted by novice nurses and endoscopists resulted in a significant drop in satisfaction (β = −10.85, *p* < 0.001). A similar pattern was observed when a novice endoscopist was paired with a competent nurse, leading to a substantial decline in satisfaction (β = −11.12, *p* < 0.001). Some combinations, such as competent endoscopists paired with expert nurses, did not yield significant differences in satisfaction compared to the baseline (*p* = 0.91).

These findings have important implications for clinical practice. The strong impact of endoscopist and nurse expertise on patient satisfaction suggests that pairing highly experienced professionals—especially in cases involving high disease activity or first-time procedures—may significantly improve the overall patient experience. These results also underscore the relevance of investing in targeted training programs and optimizing team allocation to promote patient-centered care and enhance adherence to IBD surveillance.

### 3.3. Relationship Between Patient Satisfaction and Clinical Variables

Satisfaction levels varied significantly with disease severity (χ^2^(3) = 18.92, *p* < 0.001). Post hoc analyses confirmed that patients in remission reported significantly higher satisfaction than those with severe disease (*p* < 0.001), as well as those with mild (*p* = 0.005) or moderate activity (*p* = 0.009). Furthermore, satisfaction before the exam was positively correlated with the number of years since diagnosis (r = 0.1, *p* < 0.05). A history of previous surgeries was not associated with differences in pre-exam satisfaction but was linked to lower satisfaction during the examination (U = 4.43, *p* = 0.035).

Bowel preparation methods significantly influenced satisfaction. Patients undergoing high-volume preparation reported lower satisfaction before (U = 11.62, *p* < 0.001), during (U = 14.87, *p* < 0.001), and after (U = 8.21, *p* = 0.004) the procedure, compared to those using low-volume preparation (U = 6.44, *p* = 0.011). Preparation timing also had an impact, with split-dose regimens associated with higher overall satisfaction, although the differences were not statistically significant.

Sedation type influenced the patient experience, with deep sedation linked to lower overall satisfaction (χ^2^(2) = 7.34, *p* = 0.025). Similarly, perceived bowel cleanliness affected satisfaction, with patients who reported inadequate preparation showing significantly lower satisfaction (χ^2^(2) = 6.42, *p* = 0.04, Kruskal–Wallis test).

Treatment-related factors also contributed to satisfaction variations. Patients undergoing biological therapy reported lower overall satisfaction than those not receiving biologics (U = 7.31, *p* = 0.007), while medical treatment had no significant effect. Additionally, previous colonoscopy experience played a role, with first-time patients reporting significantly lower satisfaction before (U = 7.21, *p* = 0.007), during (U = 8.04, *p* = 0.005), and after (U = 9.55, *p* = 0.002) the procedure, as well as overall (U = 10.12, *p* = 0.001).

Full statistical comparisons are available in Table 2 and Table 3.

### 3.4. Determinants and Predictors of Patient Satisfaction

The multiple regression analysis identified the key determinants of patient satisfaction, explaining 51.3% of its variance. The model demonstrated a good fit, as indicated by a Wald chi-square test (χ^2^(12) = 85.4, *p* < 0.0001). Endoscopist experience was a significant predictor, with more experienced professionals associated with higher satisfaction (β = 2.111; *p* = 0.0126). Disease activity also played a crucial role, with patients in remission reporting slightly higher satisfaction (β = 1.705; *p* = 0.0209), whereas those with severe disease exhibited markedly lower satisfaction (β = −3.872; *p* < 0.001).

The frequency of endoscopic examinations in the previous 24 months was positively associated with satisfaction (β = 0.459; *p* = 0.0413), suggesting that greater familiarity with the procedure may contribute to a more positive experience. Other clinical and procedural factors, including sedation type, preparation methods, and patient-reported stress, also contributed to variations in the satisfaction levels. The full results from the predictive model are summarized in Table 4.

## 4. Discussion

This study provides a comprehensive analysis of patient satisfaction with colonoscopy among individuals with IBD across three centers. Furthermore, the impact of procedural, psychological, and clinical factors in determining overall satisfaction with colonoscopy is investigated.

A significant observation from our study is the variation in satisfaction levels across different phases of the colonoscopy experience. Pre-examination dissatisfaction was common, likely attributed to anticipatory anxiety and the discomfort associated with bowel preparation. This aligns with previous evidence indicating that negative expectations and prior unpleasant experiences substantially influence patient attitudes toward colonoscopy [5,27]. Patients often cite bowel preparation as the most burdensome aspect of the procedure, which is further exacerbated in IBD due to heightened bowel sensitivity [28,29]. During the examination, patient satisfaction levels showed slight improvement, suggesting that procedural factors such as sedation and endoscopist expertise play a role in shaping experience. Our regression model identified physician experience as a key determinant of satisfaction, with expert-level endoscopists yielding significantly higher satisfaction scores. This is consistent with the prior literature demonstrating that patient trust and comfort increase with more experienced practitioners [27]. Moreover, effective communication from healthcare providers has been associated with reduced anxiety and increased adherence to surveillance recommendations [7,30,31]. Effective communication between healthcare providers and patients is key to colonoscopy satisfaction. Clear explanations, reassurance, and post-exam discussions help reduce anxiety and enhance the experience. Improving training for endoscopists and nurses in patient communication could further boost satisfaction and adherence to surveillance [32].

Post-examination satisfaction levels varied, with a notable increase in dissatisfaction compared to the pre-examination phase. This could be attributed to residual discomfort or delayed recovery from sedation. Moreover, dissatisfaction with procedure results could be a key to post-exam dissatisfaction. Our results indicate that patients in remission reported higher satisfaction than those with severe disease activity. This finding is particularly relevant, suggesting that disease burden and symptomatology influence patient perceptions of medical care [6]. Psychological distress, particularly anticipatory anxiety and fear of discomfort or negative results, may discourage patients from adhering to scheduled surveillance colonoscopies. Over time, this avoidance behavior can compromise the early detection of disease progression or dysplasia, ultimately affecting long-term outcomes. Those with more severe symptoms may associate colonoscopy with a distressing confirmation of disease progression, thereby negatively impacting satisfaction levels.

Bowel preparation is a key element that has emerged as strongly associated with patient satisfaction. Patients receiving low-volume preparation before, during, and after the examination show higher satisfaction than patients receiving high-volume preparation before. However, no significant benefit from the split administration of bowel preparation was detected. Clinical trials and subsequent meta-analyses have confirmed the better tolerability of low-volume bowel preparation in this field [33]. However, previous studies have also demonstrated the better tolerability and effectiveness of split bowel preparations, which did not emerge from our findings [34]. Colonoscopy remains the gold standard for IBD surveillance, but non-invasive alternatives may improve adherence. Fecal calprotectin, capsule endoscopy, and intestinal ultrasound show promise as patient-friendly options. Fecal calprotectin is non-invasive and correlates well with intestinal inflammation, but it lacks anatomical detail and may yield false positives in other gastrointestinal conditions. Capsule endoscopy offers full mucosal visualization without discomfort, yet its use is limited by cost and inability to perform biopsies. Intestinal ultrasound is patient-friendly, repeatable, and radiation-free, but it is operator-dependent and may have lower sensitivity for subtle lesions. Future research should assess their impact on satisfaction and long-term adherence [22,35].

Another key finding is the relationship between pre-exam stress levels and satisfaction. Our study demonstrated a significant negative correlation between perceived stress and patient satisfaction across all procedure phases. This aligns with prior research indicating that anxiety and stress contribute to lower tolerance and poorer overall experience during colonoscopy [5,36]. High stress levels may exacerbate perceived pain, prolong recovery, and discourage future adherence to surveillance protocols. Implementing stress-reducing interventions, such as audio or visual relaxation techniques or anxiolytic medications, could improve patient outcomes and adherence rates [37,38].

Given the strong negative correlation between perceived stress and patient satisfaction, future interventions should focus on targeted strategies to alleviate pre-exam anxiety. Psychological preparation programs, such as mindfulness-based stress reduction or cognitive behavioral therapy, have been shown to reduce procedural anxiety in other endoscopic procedures [39,40,41]. Additionally, digital support tools, including educational videos or guided relaxation apps, could enhance patient understanding and comfort before colonoscopies. For example, Broder et al. (2022) showed that short educational videos before endoscopy improved satisfaction with pre-procedural information and reduced cancelations [42]. Further studies should explore whether integrating these approaches improves adherence to surveillance guidelines and overall patient experience.

Educational level emerged as a potential predictor of satisfaction, with higher educational attainment correlating with slightly lower satisfaction scores. This may reflect heightened expectations, increased health literacy, or a more critical appraisal of medical care. These observations are consistent with prior studies suggesting that more educated patients often seek greater clarity in the communication and justification of procedures [20,43,44].

While educational level emerged as a potential predictor of satisfaction, future research should investigate additional demographic factors, such as age and socioeconomic status. Younger patients may experience higher anxiety due to unfamiliarity with the procedure, whereas older patients may face challenges related to bowel preparation [45]. Furthermore, patients with a lower socioeconomic status may have limited access to resources that facilitate preparation for and understanding of the procedure, potentially affecting their satisfaction. Addressing these disparities through personalized patient education and support could help optimize the colonoscopy experience across diverse patient populations.

Finally, our study highlights the importance of disease activity and previous colonoscopy experience in shaping patient perceptions. First-time patients reported significantly lower satisfaction scores, likely due to heightened fear and unfamiliarity with the procedure. This highlights the need for large-scale educational initiatives to address misconceptions and false myths about colonoscopy. Furthermore, seriate colonoscopy surveillance over time may improve acceptance and reduce procedural anxiety, emphasizing the role of longitudinal patient support [8]. Moreover, future research should investigate how the integration of molecular and histological markers into surveillance protocols may influence patient perceptions, satisfaction, and adherence over time.

Despite its strengths, our study has several limitations. First, the cross-sectional design limits our ability to establish causality between identified factors and patient satisfaction. Longitudinal studies would be valuable in assessing how satisfaction evolves over multiple surveillance visits. Additionally, while our sample was diverse, cultural and institutional differences across study centers may have influenced patient experiences and responses. Future research should explore these variations in a broader international context. Moreover, while our analysis accounted for key predictors, unmeasured variables such as socioeconomic status, psychological support availability, and prior healthcare experiences could further elucidate satisfaction determinants.

Furthermore, we did not specifically assess the impact of relevant comorbidities—such as cardiovascular or respiratory conditions—which may influence bowel preparation tolerance, sedation needs, and overall satisfaction. Their role should be explored in future studies to better tailor care for patients with complex clinical profiles. Similarly, the anesthetist’s role was not considered, although their expertise may also influence patient comfort and satisfaction, particularly in settings where deep sedation is used.

## 5. Conclusions

In conclusion, this study identifies the key factors influencing patient satisfaction with colonoscopy in IBD, emphasizing the impact of procedural, psychological, and clinical variables. Satisfaction levels varied across different phases, with lower scores before the procedure due to anxiety and bowel preparation discomfort. Endoscopist and nurse expertise significantly improved the experience, while disease activity influenced post-exam satisfaction. Bowel preparation quality, sedation type, and stress levels played a crucial role in shaping satisfaction. Optimizing bowel preparation, implementing stress-reducing strategies, and enhancing patient-provider communication could improve adherence to surveillance guidelines. Psychological support and tailored education for first-time patients may reduce anxiety and increase acceptance. Patients with previous colonoscopy experience reported higher satisfaction, highlighting the need for structured pre-exam education. Personalized interventions addressing stress and preparation-related discomfort may further enhance the overall experience. Future longitudinal studies should assess the long-term impact of these factors, contributing to patient-centered strategies that improve quality of care and adherence to IBD surveillance protocols. These insights may also support the integration of targeted patient education tools and guide clinical workflows aimed at improving satisfaction, adherence, and the quality of care in endoscopy practice

## Figures and Tables

**Table 1:** Characteristics of the sample.

Variables	Entire Sample (*n* = 444)
Smoker	
Yes	94 (21.2%)
No	350 (78.8%)
First Colonscopy	
Yes	92 (20.7%)
No	352 (79.3%)
Symptoms	
Yes	322 (72.5%)
No	122 (27.5%)
Previous Pathologies	
Yes	344 (77.5%)
No	100 (22.6%)
Previous Surgeries	
Yes	224 (50.5%)
No	219 (49.4%)
Stoma	
Yes	16 (3.6%)
No	428 (96.4%)
Type of Preparation	
High volume	60 (13.5%)
Low volume	384 (86.5%)
Preparation Timing	
Day before	288 (64.8%)
Split	151 (34.1%)
Same day	5 (1.1%)
Exam Completed	
Yes	367 (82.6%)
No	77 (17.3%)
Feeling of Cleanliness	
Low	36 (8.1%)
Medium	162 (36.5%)
High	246 (55.4%)
Sedation	
No	46 (10.4%)
Conscious sedation	320 (72.1%)
Deep sedation	78 (17.6%)
Nurse Experience	
Novice	56 (12.6%)
Competent	113 (25.5%)
Expert	274 (61.7%)
Endoscopist Experience	
Novice	61 (13.74%)
Competent	179 (40.32%)
Expert	204 (45.95%)
Completed Exam	444 (100%)
Disease Activity	
Remission	140 (31.53%)
Mild	104 (23.42%)
Moderate	102 (22.97%)
Severe	98 (22.07%)
Biological Therapy	
Yes	289 (65.09%)
No	155 (34.91%)
Medical Therapy	
Yes	185 (41.67%)
No	259 (58.33%)
ECSQ	
ECSQ Before [median, IQR]	15 [11.00]
ECSQ Exam [median, IQR]	11 [10.00]
ECSQ After [median, IQR]	14 [8.25]
ECSQ Total [median, IQR]	57.5 [67.50]
Preceived Stress	
PSS [median, IQR]	38 [4.00]
Low stress [n, %]	10 [2.25]
Medium stress	54 [12.2]
High stress	380 [85.6]

**Table 2:** Satisfaction and stress score comparison between groups.

	ECSQ Before Exam	ECSQ During Exam	ECSQ After Exam	ECSQ Total Score	PSS
	Median [IQR]	Median [IQR]	Median [IQR]	Median [IQR]	Median [IQR]
Sex					
Man	15 [11]	11 [10]	12 [8]	72 [45]	38 [4]
Women	15.5 [11]	10.5 [10]	14 [10]	69 [44.5]	38 [4]
Sign.	0.645	0.740	0.487	0.976	0.454
Educational Level					
Did not complete school	11 [5.5]	5 [5.5]	12 [4]	90 [25]	36 [6]
Middle school	15 [10.75]	9 [10]	12 [9.5]	86 [47]	38 [4]
High school	16 [11]	12 [10]	14 [10]	71 [46]	38 [4]
Bachelor’s degree	15 [10]	11 [9]	13 [8]	67 [42.5]	38 [4]
Sign.	0.584	0.826	0.927	0.590	0.130
Smoke					
Yes	15.5 [10]	12 [9.75]	14 [11]	76 [42]	38 [4]
No	15 [11]	10 [10]	13.5 [8]	69 [45.5]	38 [4]
Sign.	0.343	0.410	0.659	0.926	0.467
First Colonscopy					
Yes	12 [9]	8 [8]	11 [8.25]	55 [43.5]	38 [2.5]
No	16 [11]	12 [10]	15 [9]	75 [46]	38 [4]
Sign.	0.007 **	0.005 **	0.002 **	0.001 ***	0.100
Symptoms					
Yes	16 [11.75]	12 [10]	15 [9]	58 [42.5]	38 [4]
No	12 [8]	8 [7.75]	12 [7.75]	72 [46]	38 [4]
Sign.	0.001 ***	0.005 **	0.009 **	0.002 **	0.791
Previous Pathologies					
Yes	15.5 [11]	12 [10]	14 [9]	69 [41.5]	38 [4]
No	15 [10]	8 [9]	12 [8]	64 [42.5]	38 [4]
Sign.	0.236	0.033 *	0.007 **	0.126	0.321
Pathology					
Chron	15 [10]	9 [9]	12 [9]	75 [41]	38 [4]
Colitis	16 [11]	12 [9]	15 [9]	65 [42.5]	38 [4]
Sign.	0.075	0.032 *	0.002 **	0.030 *	0.284
Previous Surgeries					
Yes	15 [11]	9 [9]	12 [8]	75 [45]	38 [4]
No	16 [11]	12 [9]	14 [9.5]	85 [40]	38 [4]
Sign.	0.296	0.035	0.642	0.149	0.027 *
Stoma					
Yes	18.5 [10.25]	12 [8]	17.5 [9.25]	69 [44.5]	38 [2]
No	15 [11]	10.5 [10]	13 [9]	51 [43]	38 [4]
Sign.	0.403	0.097	0.122	0.264	0.038 *
Type of Preparation					
High volume	12 [7]	6.5 [6.25]	11 [8]	75 [46]	38 [4]
Low volume	16 [11]	12 [10]	14 [9]	53 [51]	38 [4]
Sign.	0.001 ***	0.000 ***	0.004 **	0.011 *	0.591
Preparation Timing					
Day before	15 [10]	10 [9]	13 [9]	69 [46]	38 [4]
Split	16 [11.5]	12 [10]	14 [9]	75 [42.5]	38 [4]
Same day	13 [10]	9 [9]	11 [7]	73 [51.5]	38 [0]
Sign.	0.332	0.673	0.450	0.484	0.638
Exam Completed					
Yes	15 [11]	10 [10]	13 [9.5]	64 [46]	38 [4]
No	17 [11]	12 [8]	15 [8]	55 [42.75]	38 [4]
Sign.	0.738	0.111	0.510	0.873	0.028 *
Feeling of Cleanliness					
Low	15 [12]	12 [10]	16 [7.25]	73 [51.5]	38 [2]
Medium	16 [10.75]	12 [8]	15 [9]	75 [27]	38 [4]
High	15 [10.75]	9 [9]	12 [10]	69 [46] §	38 [4]
Sign.	0.601	0.089	0.108	0.04 *	0.214
Sedation					
No	14 [10.75]	11.5 [10]	13.5 [7.75]	75 [51.5]	38 [4]
Conscious sedation	16 [11]	12 [10]	14.5 [9]	75 [48.5]	38 [4]
Deep sedation	13 [10]	8 [9]	12 [8]	64 [38] §	38 [4]
Sign.	0.918	0.326	0.101	0.025 *	0.187
Seniority of Nurse					
Novice	14 [11.25]	9.5 [10]	12.5 [9.5]	66 [49.5]	38 [4]
Competent	15 [11]	9 [9]	12 [8]	65 [40]	38 [4]
Expert	16 [11]	12 [10]	15 [9]	75 [46]	38 [4]
Sign.	0.381	0.521	0.192	0.101	0.125
Endoscopist Experience					
Novice	12 [8]	9 [8] §	12 [10]	65 [42.5]	38 [4] §
Competent	16 [11]	10 [10]	14 [9]	72 [46]	38 [4]
Expert	16 [11.25]	12 [10]	14 [8]	75 [46]	38 [4]
Sign.	0.056	0.024 *	0.295	0.373	0.009 **
Disease Activity					
Remission	20 [8.25]	14 [5.25]	17 [8]	90 [30]	38 [4]
Mild	19 [11.25]	13 [10]	16 [8]	93 [33.25]	38 [4]
Moderate	13.5 [10]	9 [8]	12 [10]	69 [45]	38 [4]
Severe	11 [3]	6 [3]	9 [4]	44 [16.5]	38 [2]
Sign.	0.005 **	0.000 ***	0.000 ***	0.000 ***	0.000 ***
Biological Therapy					
Yes	15 [10]	9 [9]	12 [9]	64 [45]	38 [4]
No	17 [11]	12 [10]	15 [9]	77 [42.5]	38 [4]
Sign.	0.037 *	0.159	0.060	0.007 **	0.470
Medical Therapy					
Yes	15 [11]	10 [9]	14 [8]	72 [45]	38 [4]
No	16 [11]	12 [10]	14 [9.5]	69 [46]	38 [4]
Sign.	0.686	0.368	0.765	0.823	0.325

Signif. codes (*p*-values): 0 ***; 0.001 **; 0.01 *; 0.05. § Difference between groups at post hoc analysis.

**Table 3:** Bivariate correlation matrix.

	Age	BMI	Years Diagnosis	Exam Duration (min)	ECSQ Before	ECSQ During	ECSQ After	ECSQ Total	PSS	Test Performed Last 24 Months (n)
Age	NA									
BMI	0.23 ***	NA								
Time from diagnosis (y)	0.2 ***	−0.02	NA							
Exam duration (min)	0.1 *	0.02	0.07	NA						
ECSQ before exam	−0.02	−0.04	0.1 *	−0.02	NA					
ECSQ intra exam	−0.05	−0.02	0.05	−0.04	0.79 ***	NA				
ECSQ after exam	−0.01	0.01	0.02	0.02	0.70 ***	0.78 ***	NA			
ECSQ total	−0.02	0	0.09	−0.01	0.84 ***	0.81 ***	0.82 ***	NA		
PSS	0.01	−0.01	0	−0.01	−0.18 ***	−0.24 ***	−0.24 ***	−0.27 ***	NA	
Test performed last 24 months (n)	−0.07	−0.04	−0.11 *	−0.24 ***	−0.02	−0.01	−0.02	−0.01	−0.02	NA

Signif. codes: 0 ***; 0.001 **; 0.01 *; 0.05.

**Table 4:** Determinants of endoscopy satisfaction.

Predictors	Estimate	SE	t-Value	*p*-Value	Sign.
Intercept	55.451	7.323	10.316	0.000	***
Middle school	−4.348	3.372	−1.290	0.197	
High school	−6.356	3.323	−1.913	0.056	.
Bachelor’s degree	−6.389	3.324	−1.922	0.055	.
Competent level endoscopist	0.995	0.859	1.158	0.247	
**Expert level endoscopist**	**2.111**	**0.843**	**2.504**	**0.012**	*
**Mild disease activity**	**1.705**	**0.735**	**2.318**	**0.020**	*
Moderate disease activity	−0.267	0.764	−0.349	0.727	
**Severe disease activity**	**−3.872**	**0.862**	**−4.492**	**0.000**	***
**Tests performed in the last 24 months (n)**	**0.459**	**0.224**	**2.046**	**0.041**	*
Sedation	−4.203	2.917	−1.441	0.150	
Medical therapy	3.053	3.118	0.979	0.328	
Biological therapy	4.641	3.355	1.383	0.167	

Signif. codes: 0 ***; 0.001 **; 0.01 *; 0.05; Bolded values represent the statistically significant predictors.

## Data Availability

Data can be provided by the first author upon reasonable request.

## Supplementary Materials

The following supporting information can be downloaded at: https://www.mdpi.com/article/10.3390/jcm14082562/s1, Table S1: Socio-demographic characteristics.
