# Supplementary material for: Patient Satisfaction with IBD Undergoing Colonoscopy: A Multicenter Cross-Sectional Study
Source: J Clin Med. 2025 Apr 8;14(8):2562. doi: 10.3390/jcm14082562 (PMC12027919; doi:10.3390/jcm14082562)
Supplement: Supplementary file 1 [file jcm-14-02562-s001.zip › jcm-3552854-supplementary.pdf]

Table S1. Socio-demographic characteristics

Variables	Entire Sample (n=444)	Chron (n=251)	Colitis (n=203)	Differences
Age [Median, IQR]	53 [25.25]	52 [23]	54 [28.5]	—
BMI [Median, IQR]	24 [6.00]	24 [6]	24 [6]	—
Years from diagnosis [Median, IQR]	8 [13.00]	7 [13]	8 [13]	—
Sex				
Male [n, %]	244, (54.9%)	129 (53.5%)	115 (56.6%)	—
Female [n, %]	200, (45.1%)	112 (46.5%)	88 (43.4%)	—
Nationality				
Italian	433 (97.5%)	236 (97.9%)	197 (97%)	—
Educational level				
Did not complete school	3 (0.7%)	1 (0.41%)	2 (0.99%)	—
Middle school	66 (14.8%)	35 (14.5%)	31 (15.3%)	—
High school	212 (47.7%)	116 (48.1%)	96 (47.3%)	—
Bachelor's degree	163 (36.7%)	89 (36.9%)	74 (36.4%)	—
